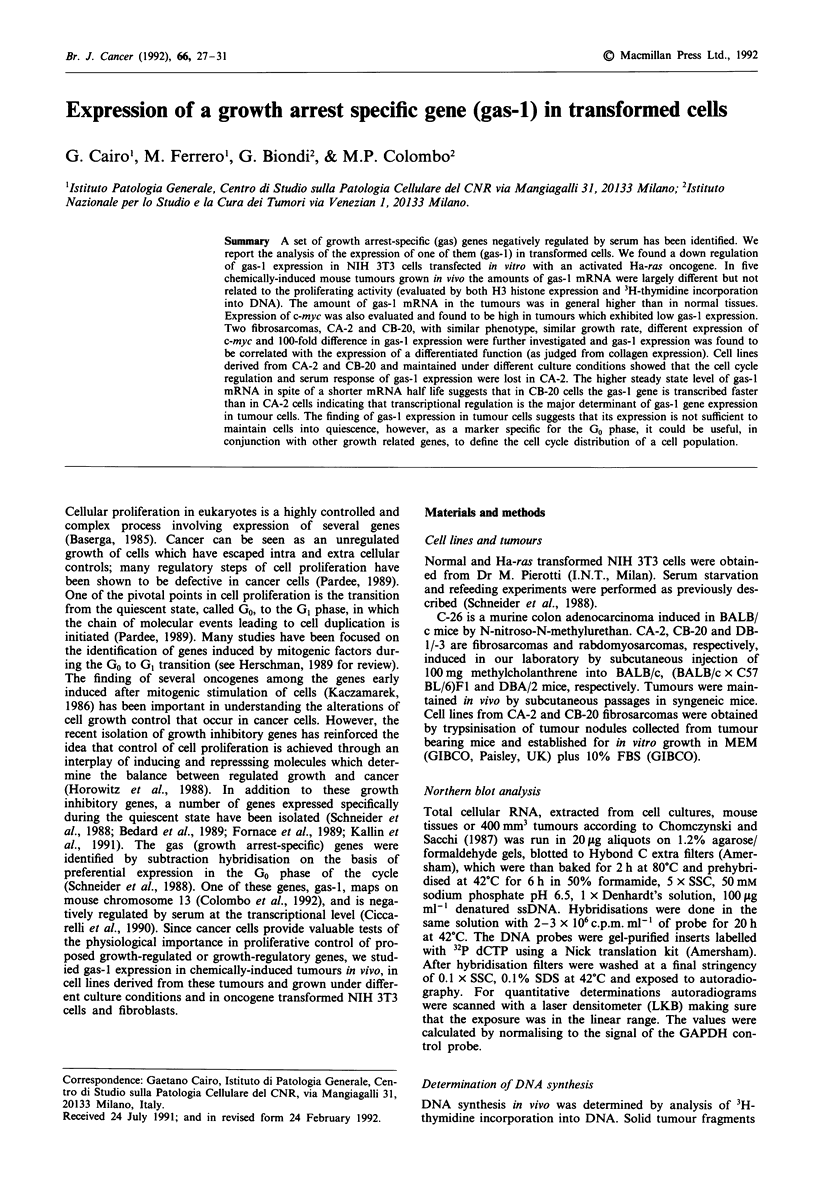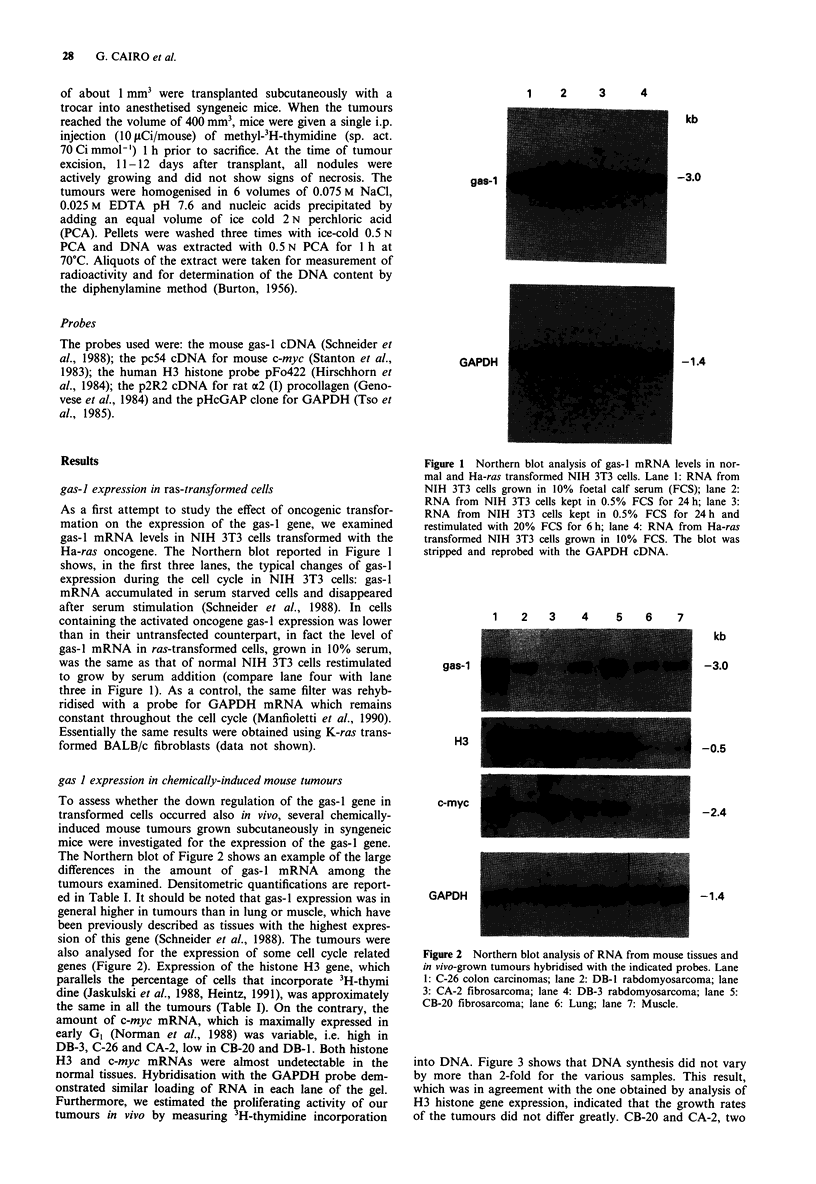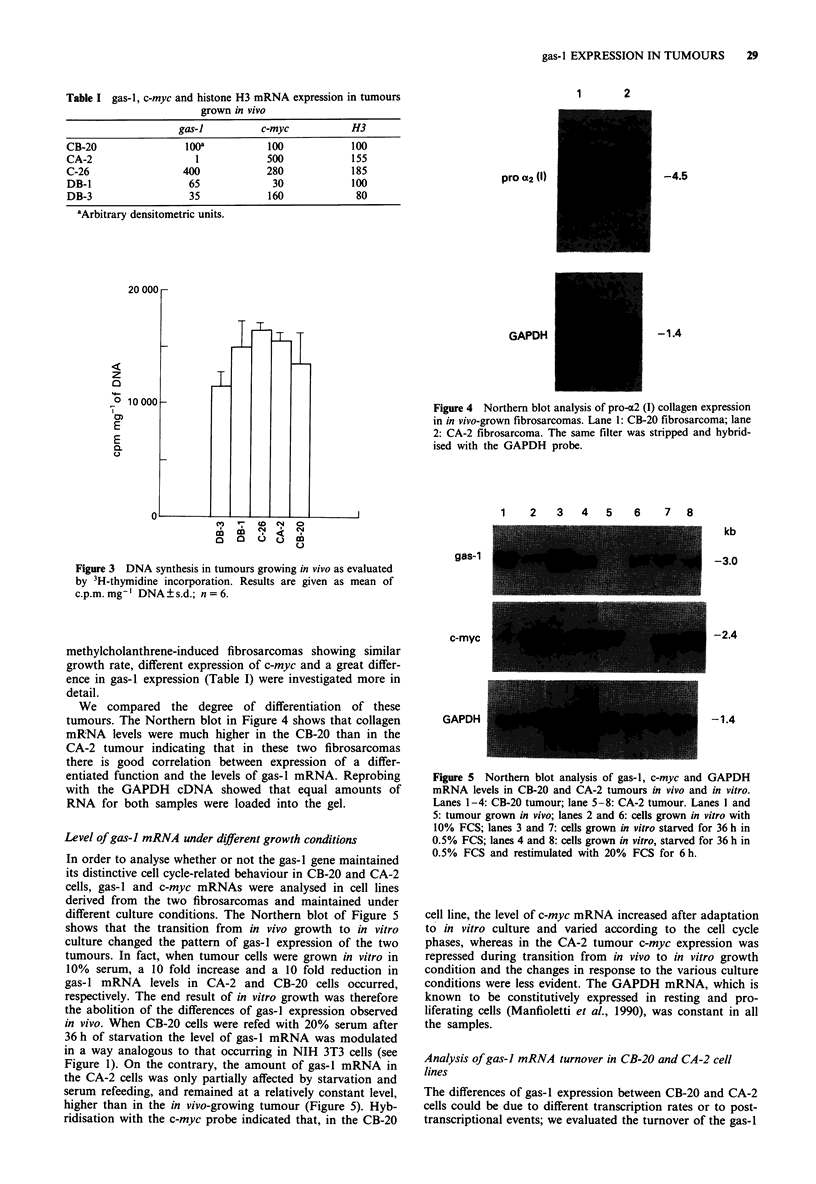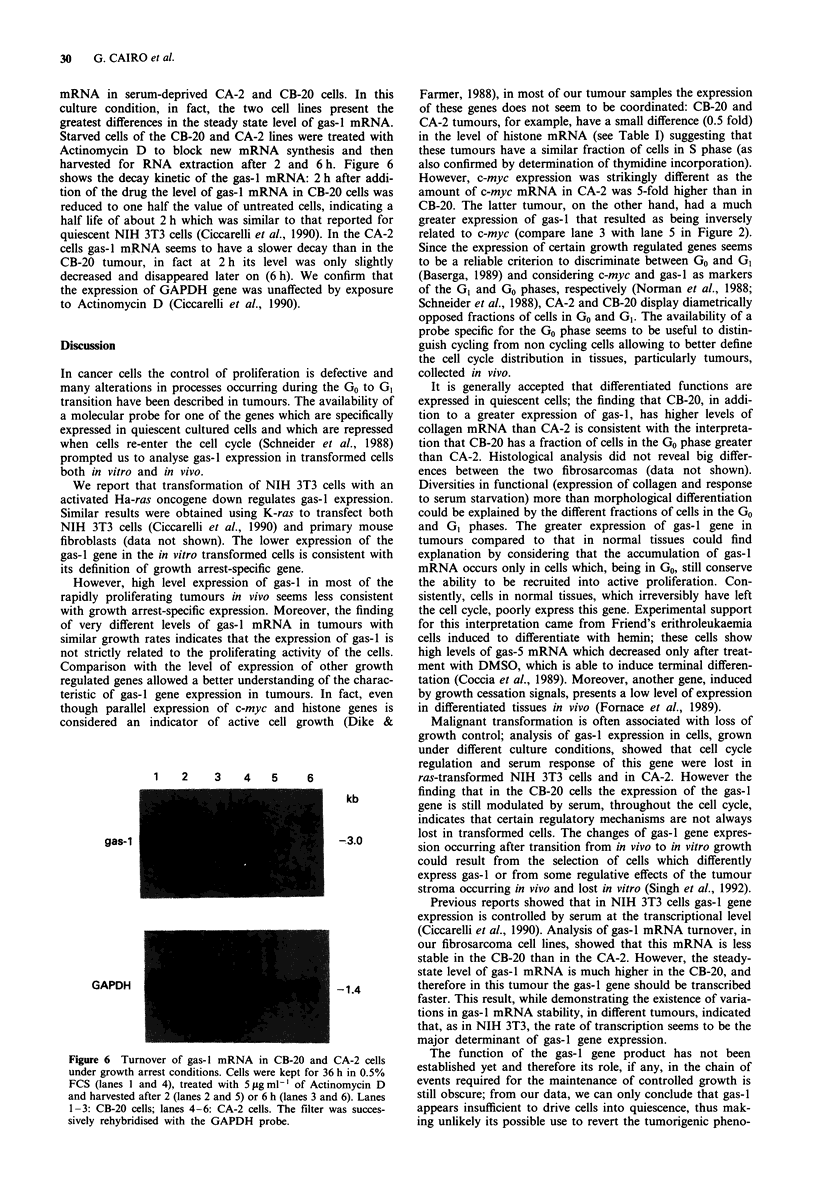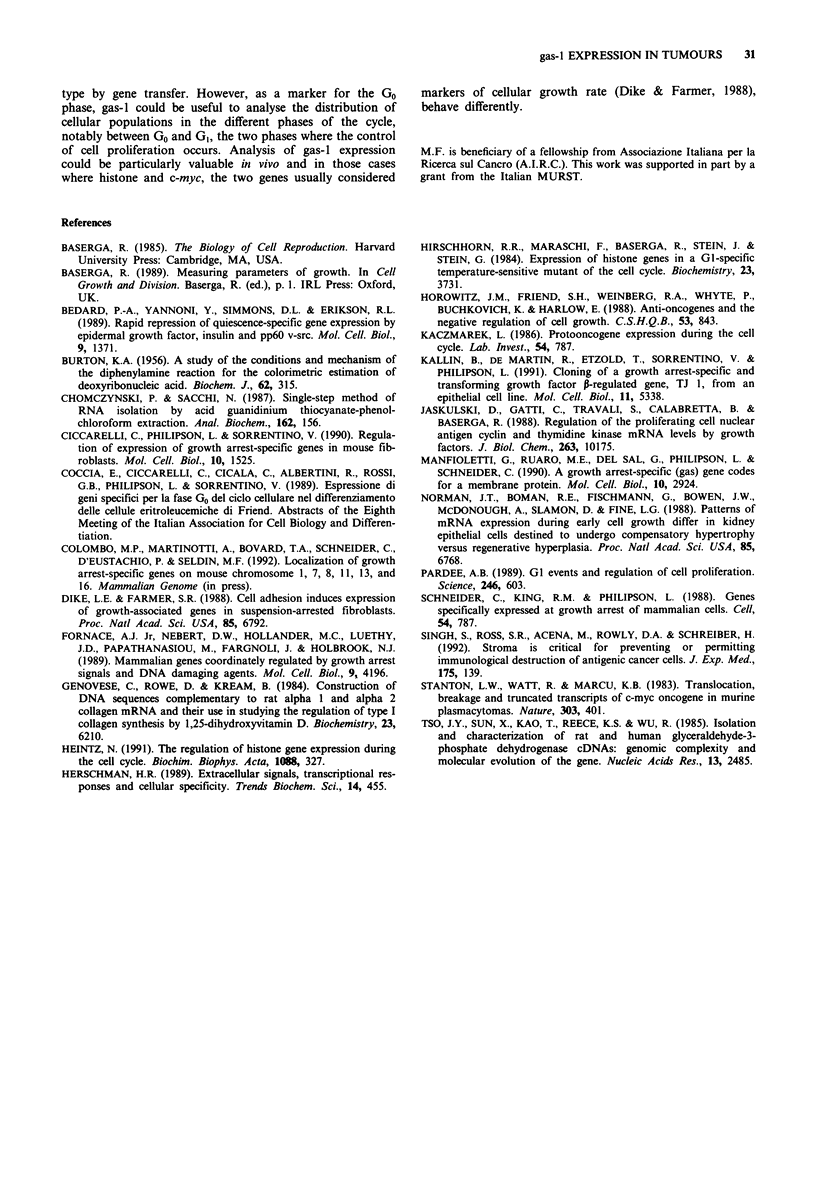# Expression of a growth arrest specific gene (gas-1) in transformed cells.

**DOI:** 10.1038/bjc.1992.211

**Published:** 1992-07

**Authors:** G. Cairo, M. Ferrero, G. Biondi, M. P. Colombo

**Affiliations:** Istituto Patologia Generale, Centro di Studio sulla Patologia Cellulare del CNR, Milano, Italy.

## Abstract

**Images:**


*Br. J. Cancer* (1992), **66**, 27–31 © Macmillan Press Ltd., 1992

### Expression of a growth arrest specific gene (gas-1) in transformed cells

G. Cairo[1], M. Ferrero[1], G. Biondi[2], & M.P. Colombo[2]

[1]*Istituto Patologia Generale, Centro di Studio sulla Patologia Cellulare del CNR via Mangiagalli 31, 20133 Milano;* [2]*Istituto Nazionale per lo Studio e la Cura dei Tumori via Venezian 1, 20133 Milano.*

**Summary** A set of growth arrest-specific (gas) genes negatively regulated by serum has been identified. We report the analysis of the expression of one of them (gas-1) in transformed cells. We found a down regulation of gas-1 expression in NIH 3T3 cells transfected *in vitro* with an activated Ha-*ras* oncogene. In five chemically-induced mouse tumours grown *in vivo* the amounts of gas-1 mRNA were largely different but not related to the proliferating activity (evaluated by both H3 histone expression and $^3$H-thymidine incorporation into DNA). The amount of gas-1 mRNA in the tumours was in general higher than in normal tissues. Expression of c-*myc* was also evaluated and found to be high in tumours which exhibited low gas-1 expression. Two fibrosarcomas, CA-2 and CB-20, with similar phenotype, similar growth rate, different expression of c-*myc* and 100-fold difference in gas-1 expression were further investigated and gas-1 expression was found to be correlated with the expression of a differentiated function (as judged from collagen expression). Cell lines derived from CA-2 and CB-20 and maintained under different culture conditions showed that the cell cycle regulation and serum response of gas-1 expression were lost in CA-2. The higher steady state level of gas-1 mRNA in spite of a shorter mRNA half life suggests that in CB-20 cells the gas-1 gene is transcribed faster than in CA-2 cells indicating that transcriptional regulation is the major determinant of gas-1 gene expression in tumour cells. The finding of gas-1 expression in tumour cells suggests that its expression is not sufficient to maintain cells into quiescence, however, as a marker specific for the $G_0$ phase, it could be useful, in conjunction with other growth related genes, to define the cell cycle distribution of a cell population.

Cellular proliferation in eukaryotes is a highly controlled and complex process involving expression of several genes (Baserga, 1985). Cancer can be seen as an unregulated growth of cells which have escaped intra and extra cellular controls; many regulatory steps of cell proliferation have been shown to be defective in cancer cells (Pardee, 1989). One of the pivotal points in cell proliferation is the transition from the quiescent state, called $G_0$, to the $G_1$ phase, in which the chain of molecular events leading to cell duplication is initiated (Pardee, 1989). Many studies have been focused on the identification of genes induced by mitogenic factors during the $G_0$ to $G_1$ transition (see Herschman, 1989 for review). The finding of several oncogenes among the genes early induced after mitogenic stimulation of cells (Kaczamarek, 1986) has been important in understanding the alterations of cell growth control that occur in cancer cells. However, the recent isolation of growth inhibitory genes has reinforced the idea that control of cell proliferation is achieved through an interplay of inducing and represssing molecules which determine the balance between regulated growth and cancer (Horowitz *et al.*, 1988). In addition to these growth inhibitory genes, a number of genes expressed specifically during the quiescent state have been isolated (Schneider *et al.*, 1988; Bedard *et al.*, 1989; Fornace *et al.*, 1989; Kallin *et al.*, 1991). The gas (growth arrest-specific) genes were identified by subtraction hybridisation on the basis of preferential expression in the $G_0$ phase of the cycle (Schneider *et al.*, 1988). One of these genes, gas-1, maps on mouse chromosome 13 (Colombo *et al.*, 1992), and is negatively regulated by serum at the transcriptional level (Ciccarelli *et al.*, 1990). Since cancer cells provide valuable tests of the physiological importance in proliferative control of proposed growth-regulated or growth-regulatory genes, we studied gas-1 expression in chemically-induced tumours *in vivo*, in cell lines derived from these tumours and grown under different culture conditions and in oncogene transformed NIH 3T3 cells and fibroblasts.

Correspondence: Gaetano Cairo, Istituto di Patologia Generale, Centro di Studio sulla Patologia Cellulare del CNR, via Mangiagalli 31, 20133 Milano, Italy.
Received 24 July 1991; and in revised form 24 February 1992.

### Materials and methods

### *Cell lines and tumours*

Normal and Ha-*ras* transformed NIH 3T3 cells were obtained from Dr M. Pierotti (I.N.T., Milan). Serum starvation and refeeding experiments were performed as previously described (Schneider *et al.*, 1988).

C-26 is a murine colon adenocarcinoma induced in BALB/c mice by N-nitroso-N-methylurethan. CA-2, CB-20 and DB-1/-3 are fibrosarcomas and rabdomyosarcomas, respectively, induced in our laboratory by subcutaneous injection of 100 mg methylcholanthrene into BALB/c, (BALB/c × C57 BL/6)F1 and DBA/2 mice, respectively. Tumours were maintained *in vivo* by subcutaneous passages in syngeneic mice. Cell lines from CA-2 and CB-20 fibrosarcomas were obtained by trypsinisation of tumour nodules collected from tumour bearing mice and established for *in vitro* growth in MEM (GIBCO, Paisley, UK) plus 10% FBS (GIBCO).

### *Northern blot analysis*

Total cellular RNA, extracted from cell cultures, mouse tissues or 400 $mm^3$ tumours according to Chomczynski and Sacchi (1987) was run in 20 $\mu$g aliquots on 1.2% agarose/formaldehyde gels, blotted to Hybond C extra filters (Amersham), which were than baked for 2 h at 80°C and prehybridised at 42°C for 6 h in 50% formamide, 5 × SSC, 50 mM sodium phosphate pH 6.5, 1 × Denhardt's solution, 100 $\mu$g $ml^{-1}$ denatured ssDNA. Hybridisations were done in the same solution with $2-3 \times 10^6$ c.p.m. $ml^{-1}$ of probe for 20 h at 42°C. The DNA probes were gel-purified inserts labelled with $^{32}$P dCTP using a Nick translation kit (Amersham). After hybridisation filters were washed at a final stringency of 0.1 × SSC, 0.1% SDS at 42°C and exposed to autoradiography. For quantitative determinations autoradiograms were scanned with a laser densitometer (LKB) making sure that the exposure was in the linear range. The values were calculated by normalising to the signal of the GAPDH control probe.

### *Determination of DNA synthesis*

DNA synthesis *in vivo* was determined by analysis of $^3$H-thymidine incorporation into DNA. Solid tumour fragments of about 1 mm$^3$ were transplanted subcutaneously with a trocar into anesthetised syngeneic mice. When the tumours reached the volume of 400 mm$^3$, mice were given a single i.p. injection (10 $\mu$Ci/mouse) of methyl-$^3$H-thymidine (sp. act. 70 Ci mmol$^{-1}$) 1 h prior to sacrifice. At the time of tumour excision, 11–12 days after transplant, all nodules were actively growing and did not show signs of necrosis. The tumours were homogenised in 6 volumes of 0.075 M NaCl, 0.025 M EDTA pH 7.6 and nucleic acids precipitated by adding an equal volume of ice cold 2 N perchloric acid (PCA). Pellets were washed three times with ice-cold 0.5 N PCA and DNA was extracted with 0.5 N PCA for 1 h at 70°C. Aliquots of the extract were taken for measurement of radioactivity and for determination of the DNA content by the diphenylamine method (Burton, 1956).

*Probes*

The probes used were: the mouse gas-1 cDNA (Schneider *et al.*, 1988); the pc54 cDNA for mouse c-*myc* (Stanton *et al.*, 1983); the human H3 histone probe pFo422 (Hirschhorn *et al.*, 1984); the p2R2 cDNA for rat α2 (I) procollagen (Genovese *et al.*, 1984) and the pHcGAP clone for GAPDH (Tso *et al.*, 1985).

### Results

*gas-1 expression in ras-transformed cells*

As a first attempt to study the effect of oncogenic transformation on the expression of the gas-1 gene, we examined gas-1 mRNA levels in NIH 3T3 cells transformed with the Ha-*ras* oncogene. The Northern blot reported in Figure 1 shows, in the first three lanes, the typical changes of gas-1 expression during the cell cycle in NIH 3T3 cells: gas-1 mRNA accumulated in serum starved cells and disappeared after serum stimulation (Schneider *et al.*, 1988). In cells containing the activated oncogene gas-1 expression was lower than in their untransfected counterpart, in fact the level of gas-1 mRNA in *ras*-transformed cells, grown in 10% serum, was the same as that of normal NIH 3T3 cells restimulated to grow by serum addition (compare lane four with lane three in Figure 1). As a control, the same filter was rehybridised with a probe for GAPDH mRNA which remains constant throughout the cell cycle (Manfioletti *et al.*, 1990). Essentially the same results were obtained using K-*ras* transformed BALB/c fibroblasts (data not shown).

*gas 1 expression in chemically-induced mouse tumours*

To assess whether the down regulation of the gas-1 gene in transformed cells occurred also *in vivo*, several chemically-induced mouse tumours grown subcutaneously in syngeneic mice were investigated for the expression of the gas-1 gene. The Northern blot of Figure 2 shows an example of the large differences in the amount of gas-1 mRNA among the tumours examined. Densitometric quantifications are reported in Table I. It should be noted that gas-1 expression was in general higher in tumours than in lung or muscle, which have been previously described as tissues with the highest expression of this gene (Schneider *et al.*, 1988). The tumours were also analysed for the expression of some cell cycle related genes (Figure 2). Expression of the histone H3 gene, which parallels the percentage of cells that incorporate $^3$H-thymi dine (Jaskulski *et al.*, 1988, Heintz, 1991), was approximately the same in all the tumours (Table I). On the contrary, the amount of c-*myc* mRNA, which is maximally expressed in early $G_1$ (Norman *et al.*, 1988) was variable, i.e. high in DB-3, C-26 and CA-2, low in CB-20 and DB-1. Both histone H3 and c-*myc* mRNAs were almost undetectable in the normal tissues. Hybridisation with the GAPDH probe demonstrated similar loading of RNA in each lane of the gel. Furthermore, we estimated the proliferating activity of our tumours *in vivo* by measuring $^3$H-thymidine incorporation into DNA. Figure 3 shows that DNA synthesis did not vary by more than 2-fold for the various samples. This result, which was in agreement with the one obtained by analysis of H3 histone gene expression, indicated that the growth rates of the tumours did not differ greatly. CB-20 and CA-2, two

**Figure 1** Northern blot analysis of gas-1 mRNA levels in normal and Ha-*ras* transformed NIH 3T3 cells. Lane 1: RNA from NIH 3T3 cells grown in 10% foetal calf serum (FCS); lane 2: RNA from NIH 3T3 cells kept in 0.5% FCS for 24 h; lane 3: RNA from NIH 3T3 cells kept in 0.5% FCS for 24 h and restimulated with 20% FCS for 6 h; lane 4: RNA from Ha-*ras* transformed NIH 3T3 cells grown in 10% FCS. The blot was stripped and reprobed with the GAPDH cDNA.

**Figure 2** Northern blot analysis of RNA from mouse tissues and *in vivo*-grown tumours hybridised with the indicated probes. Lane 1: C-26 colon carcinomas; lane 2: DB-1 rabdomyosarcoma; lane 3: CA-2 fibrosarcoma; lane 4: DB-3 rabdomyosarcoma; lane 5: CB-20 fibrosarcoma; lane 6: Lung; lane 7: Muscle.

**Table I** gas-1, c-*myc* and histone H3 mRNA expression in tumours grown *in vivo*

| | *gas-1* | *c-myc* | *H3* |
|---|---|---|---|
| CB-20 | 100[a] | 100 | 100 |
| CA-2 | 1 | 500 | 155 |
| C-26 | 400 | 280 | 185 |
| DB-1 | 65 | 30 | 100 |
| DB-3 | 35 | 160 | 80 |

[a]Arbitrary densitometric units.

**Figure 3** DNA synthesis in tumours growing *in vivo* as evaluated by $^{3}$H-thymidine incorporation. Results are given as mean of c.p.m. mg$^{-1}$ DNA±s.d.; $n = 6$.

methylcholanthrene-induced fibrosarcomas showing similar growth rate, different expression of c-*myc* and a great difference in gas-1 expression (Table I) were investigated more in detail.

We compared the degree of differentiation of these tumours. The Northern blot in Figure 4 shows that collagen mRNA levels were much higher in the CB-20 than in the CA-2 tumour indicating that in these two fibrosarcomas there is good correlation between expression of a differentiated function and the levels of gas-1 mRNA. Reprobing with the GAPDH cDNA showed that equal amounts of RNA for both samples were loaded into the gel.

**Figure 4** Northern blot analysis of pro-α2 (I) collagen expression in *in vivo*-grown fibrosarcomas. Lane 1: CB-20 fibrosarcoma; lane 2: CA-2 fibrosarcoma. The same filter was stripped and hybridised with the GAPDH probe.

**Figure 5** Northern blot analysis of gas-1, c-*myc* and GAPDH mRNA levels in CB-20 and CA-2 tumours *in vivo* and *in vitro*. Lanes 1–4: CB-20 tumour; lane 5–8: CA-2 tumour. Lanes 1 and 5: tumour grown *in vivo*; lanes 2 and 6: cells grown *in vitro* with 10% FCS; lanes 3 and 7: cells grown *in vitro* starved for 36 h in 0.5% FCS; lanes 4 and 8: cells grown *in vitro*, starved for 36 h in 0.5% FCS and restimulated with 20% FCS for 6 h.

*Level of gas-1 mRNA under different growth conditions*

In order to analyse whether or not the gas-1 gene maintained its distinctive cell cycle-related behaviour in CB-20 and CA-2 cells, gas-1 and c-*myc* mRNAs were analysed in cell lines derived from the two fibrosarcomas and maintained under different culture conditions. The Northern blot of Figure 5 shows that the transition from *in vivo* growth to *in vitro* culture changed the pattern of gas-1 expression of the two tumours. In fact, when tumour cells were grown *in vitro* in 10% serum, a 10 fold increase and a 10 fold reduction in gas-1 mRNA levels in CA-2 and CB-20 cells occurred, respectively. The end result of *in vitro* growth was therefore the abolition of the differences of gas-1 expression observed *in vivo*. When CB-20 cells were refed with 20% serum after 36 h of starvation the level of gas-1 mRNA was modulated in a way analogous to that occurring in NIH 3T3 cells (see Figure 1). On the contrary, the amount of gas-1 mRNA in the CA-2 cells was only partially affected by starvation and serum refeeding, and remained at a relatively constant level, higher than in the *in vivo*-growing tumour (Figure 5). Hybridisation with the c-*myc* probe indicated that, in the CB-20 cell line, the level of c-*myc* mRNA increased after adaptation to *in vitro* culture and varied according to the cell cycle phases, whereas in the CA-2 tumour c-*myc* expression was repressed during transition from *in vivo* to *in vitro* growth condition and the changes in response to the various culture conditions were less evident. The GAPDH mRNA, which is known to be constitutively expressed in resting and proliferating cells (Manfioletti *et al.*, 1990), was constant in all the samples.

*Analysis of gas-1 mRNA turnover in CB-20 and CA-2 cell lines*

The differences of gas-1 expression between CB-20 and CA-2 cells could be due to different transcription rates or to post-transcriptional events; we evaluated the turnover of the gas-1

mRNA in serum-deprived CA-2 and CB-20 cells. In this culture condition, in fact, the two cell lines present the greatest differences in the steady state level of gas-1 mRNA. Starved cells of the CB-20 and CA-2 lines were treated with Actinomycin D to block new mRNA synthesis and then harvested for RNA extraction after 2 and 6 h. Figure 6 shows the decay kinetic of the gas-1 mRNA: 2 h after addition of the drug the level of gas-1 mRNA in CB-20 cells was reduced to one half the value of untreated cells, indicating a half life of about 2 h which was similar to that reported for quiescent NIH 3T3 cells (Ciccarelli *et al.*, 1990). In the CA-2 cells gas-1 mRNA seems to have a slower decay than in the CB-20 tumour, in fact at 2 h its level was only slightly decreased and disappeared later on (6 h). We confirm that the expression of GAPDH gene was unaffected by exposure to Actinomycin D (Ciccarelli *et al.*, 1990).

### Discussion

In cancer cells the control of proliferation is defective and many alterations in processes occurring during the $G_0$ to $G_1$ transition have been described in tumours. The availability of a molecular probe for one of the genes which are specifically expressed in quiescent cultured cells and which are repressed when cells re-enter the cell cycle (Schneider *et al.*, 1988) prompted us to analyse gas-1 expression in transformed cells both *in vitro* and *in vivo*.

We report that transformation of NIH 3T3 cells with an activated Ha-*ras* oncogene down regulates gas-1 expression. Similar results were obtained using K-*ras* to transfect both NIH 3T3 cells (Ciccarelli *et al.*, 1990) and primary mouse fibroblasts (data not shown). The lower expression of the gas-1 gene in the *in vitro* transformed cells is consistent with its definition of growth arrest-specific gene.

However, high level expression of gas-1 in most of the rapidly proliferating tumours *in vivo* seems less consistent with growth arrest-specific expression. Moreover, the finding of very different levels of gas-1 mRNA in tumours with similar growth rates indicates that the expression of gas-1 is not strictly related to the proliferating activity of the cells. Comparison with the level of expression of other growth regulated genes allowed a better understanding of the characteristic of gas-1 gene expression in tumours. In fact, even though parallel expression of c-*myc* and histone genes is considered an indicator of active cell growth (Dike & Farmer, 1988), in most of our tumour samples the expression of these genes does not seem to be coordinated: CB-20 and CA-2 tumours, for example, have a small difference (0.5 fold) in the level of histone mRNA (see Table I) suggesting that these tumours have a similar fraction of cells in S phase (as also confirmed by determination of thymidine incorporation). However, c-*myc* expression was strikingly different as the amount of c-*myc* mRNA in CA-2 was 5-fold higher than in CB-20. The latter tumour, on the other hand, had a much greater expression of gas-1 that resulted as being inversely related to c-*myc* (compare lane 3 with lane 5 in Figure 2). Since the expression of certain growth regulated genes seems to be a reliable criterion to discriminate between $G_0$ and $G_1$ (Baserga, 1989) and considering c-*myc* and gas-1 as markers of the $G_1$ and $G_0$ phases, respectively (Norman *et al.*, 1988; Schneider *et al.*, 1988), CA-2 and CB-20 display diametrically opposed fractions of cells in $G_0$ and $G_1$. The availability of a probe specific for the $G_0$ phase seems to be useful to distinguish cycling from non cycling cells allowing to better define the cell cycle distribution in tissues, particularly tumours, collected *in vivo*.

**Figure 6** Turnover of gas-1 mRNA in CB-20 and CA-2 cells under growth arrest conditions. Cells were kept for 36 h in 0.5% FCS (lanes 1 and 4), treated with 5 $\mu$g ml$^{-1}$ of Actinomycin D and harvested after 2 (lanes 2 and 5) or 6 h (lanes 3 and 6). Lanes 1–3: CB-20 cells; lanes 4–6: CA-2 cells. The filter was successively rehybridised with the GAPDH probe.

It is generally accepted that differentiated functions are expressed in quiescent cells; the finding that CB-20, in addition to a greater expression of gas-1, has higher levels of collagen mRNA than CA-2 is consistent with the interpretation that CB-20 has a fraction of cells in the $G_0$ phase greater than CA-2. Histological analysis did not reveal big differences between the two fibrosarcomas (data not shown). Diversities in functional (expression of collagen and response to serum starvation) more than morphological differentiation could be explained by the different fractions of cells in the $G_0$ and $G_1$ phases. The greater expression of gas-1 gene in tumours compared to that in normal tissues could find explanation by considering that the accumulation of gas-1 mRNA occurs only in cells which, being in $G_0$, still conserve the ability to be recruited into active proliferation. Consistently, cells in normal tissues, which irreversibly have left the cell cycle, poorly express this gene. Experimental support for this interpretation came from Friend's erithroleukaemia cells induced to differentiate with hemin; these cells show high levels of gas-5 mRNA which decreased only after treatment with DMSO, which is able to induce terminal differentation (Coccia *et al.*, 1989). Moreover, another gene, induced by growth cessation signals, presents a low level of expression in differentiated tissues *in vivo* (Fornace *et al.*, 1989).

Malignant transformation is often associated with loss of growth control; analysis of gas-1 expression in cells, grown under different culture conditions, showed that cell cycle regulation and serum response of this gene were lost in *ras*-transformed NIH 3T3 cells and in CA-2. However the finding that in the CB-20 cells the expression of the gas-1 gene is still modulated by serum, throughout the cell cycle, indicates that certain regulatory mechanisms are not always lost in transformed cells. The changes of gas-1 gene expression occurring after transition from *in vivo* to *in vitro* growth could result from the selection of cells which differently express gas-1 or from some regulative effects of the tumour stroma occurring *in vivo* and lost *in vitro* (Singh *et al.*, 1992).

Previous reports showed that in NIH 3T3 cells gas-1 gene expression is controlled by serum at the transcriptional level (Ciccarelli *et al.*, 1990). Analysis of gas-1 mRNA turnover, in our fibrosarcoma cell lines, showed that this mRNA is less stable in the CB-20 than in the CA-2. However, the steady-state level of gas-1 mRNA is much higher in the CB-20, and therefore in this tumour the gas-1 gene should be transcribed faster. This result, while demonstrating the existence of variations in gas-1 mRNA stability, in different tumours, indicated that, as in NIH 3T3, the rate of transcription seems to be the major determinant of gas-1 gene expression.

The function of the gas-1 gene product has not been established yet and therefore its role, if any, in the chain of events required for the maintenance of controlled growth is still obscure; from our data, we can only conclude that gas-1 appears insufficient to drive cells into quiescence, thus making unlikely its possible use to revert the tumorigenic phenotype by gene transfer. However, as a marker for the $G_0$ phase, gas-1 could be useful to analyse the distribution of cellular populations in the different phases of the cycle, notably between $G_0$ and $G_1$, the two phases where the control of cell proliferation occurs. Analysis of gas-1 expression could be particularly valuable *in vivo* and in those cases where histone and c-*myc*, the two genes usually considered markers of cellular growth rate (Dike & Farmer, 1988), behave differently.

M.F. is beneficiary of a fellowship from Associazione Italiana per la Ricerca sul Cancro (A.I.R.C.). This work was supported in part by a grant from the Italian MURST.

### References

BASERGA, R. (1985). *The Biology of Cell Reproduction*. Harvard University Press: Cambridge, MA, USA.

BASERGA, R. (1989). Measuring parameters of growth. In *Cell Growth and Division*. Baserga, R. (ed.), p. 1. IRL Press: Oxford, UK.

BEDARD, P.-A., YANNONI, Y., SIMMONS, D.L. & ERIKSON, R.L. (1989). Rapid repression of quiescence-specific gene expression by epidermal growth factor, insulin and pp60 v-src. *Mol. Cell. Biol.*, **9**, 1371.

BURTON, K.A. (1956). A study of the conditions and mechanism of the diphenylamine reaction for the colorimetric estimation of deoxyribonucleic acid. *Biochem. J.*, **62**, 315.

CHOMCZYNSKI, P. & SACCHI, N. (1987). Single-step method of RNA isolation by acid guanidinium thiocyanate-phenol-chloroform extraction. *Anal. Biochem.*, **162**, 156.

CICCARELLI, C., PHILIPSON, L. & SORRENTINO, V. (1990). Regulation of expression of growth arrest-specific genes in mouse fibroblasts. *Mol. Cell. Biol.*, **10**, 1525.

COCCIA, E., CICCARELLI, C., CICALA, C., ALBERTINI, R., ROSSI, G.B., PHILIPSON, L. & SORRENTINO, V. (1989). Espressione di geni specifici per la fase $G_0$ del ciclo cellulare nel differenziamento delle cellule eritroleucemiche di Friend. Abstracts of the Eighth Meeting of the Italian Association for Cell Biology and Differentiation.

COLOMBO, M.P., MARTINOTTI, A., BOVARD, T.A., SCHNEIDER, C., D'EUSTACHIO, P. & SELDIN, M.F. (1992). Localization of growth arrest-specific genes on mouse chromosome 1, 7, 8, 11, 13, and 16. *Mammalian Genome* (in press).

DIKE, L.E. & FARMER, S.R. (1988). Cell adhesion induces expression of growth-associated genes in suspension-arrested fibroblasts. *Proc. Natl Acad. Sci. USA*, **85**, 6792.

FORNACE, A.J. Jr, NEBERT, D.W., HOLLANDER, M.C., LUETHY, J.D., PAPATHANASIOU, M., FARGNOLI, J. & HOLBROOK, N.J. (1989). Mammalian genes coordinately regulated by growth arrest signals and DNA damaging agents. *Mol. Cell. Biol.*, **9**, 4196.

GENOVESE, C., ROWE, D. & KREAM, B. (1984). Construction of DNA sequences complementary to rat alpha 1 and alpha 2 collagen mRNA and their use in studying the regulation of type I collagen synthesis by 1,25-dihydroxyvitamin D. *Biochemistry*, **23**, 6210.

HEINTZ, N. (1991). The regulation of histone gene expression during the cell cycle. *Biochim. Biophys. Acta*, **1088**, 327.

HERSCHMAN, H.R. (1989). Extracellular signals, transcriptional responses and cellular specificity. *Trends Biochem. Sci.*, **14**, 455.

HIRSCHHORN, R.R., MARASCHI, F., BASERGA, R., STEIN, J. & STEIN, G. (1984). Expression of histone genes in a G1-specific temperature-sensitive mutant of the cell cycle. *Biochemistry*, **23**, 3731.

HOROWITZ, J.M., FRIEND, S.H., WEINBERG, R.A., WHYTE, P., BUCHKOVICH, K. & HARLOW, E. (1988). Anti-oncogenes and the negative regulation of cell growth. *C.S.H.Q.B.*, **53**, 843.

KACZMAREK, L. (1986). Protooncogene expression during the cell cycle. *Lab. Invest.*, **54**, 787.

KALLIN, B., DE MARTIN, R., ETZOLD, T., SORRENTINO, V. & PHILIPSON, L. (1991). Cloning of a growth arrest-specific and transforming growth factor β-regulated gene, TJ 1, from an epithelial cell line. *Mol. Cell. Biol.*, **11**, 5338.

JASKULSKI, D., GATTI, C., TRAVALI, S., CALABRETTA, B. & BASERGA, R. (1988). Regulation of the proliferating cell nuclear antigen cyclin and thymidine kinase mRNA levels by growth factors. *J. Biol. Chem.*, **263**, 10175.

MANFIOLETTI, G., RUARO, M.E., DEL SAL, G., PHILIPSON, L. & SCHNEIDER, C. (1990). A growth arrest-specific (gas) gene codes for a membrane protein. *Mol. Cell. Biol.*, **10**, 2924.

NORMAN, J.T., BOMAN, R.E., FISCHMANN, G., BOWEN, J.W., McDONOUGH, A., SLAMON, D. & FINE, L.G. (1988). Patterns of mRNA expression during early cell growth differ in kidney epithelial cells destined to undergo compensatory hypertrophy versus regenerative hyperplasia. *Proc. Natl Acad. Sci. USA*, **85**, 6768.

PARDEE, A.B. (1989). G1 events and regulation of cell proliferation. *Science*, **246**, 603.

SCHNEIDER, C., KING, R.M. & PHILIPSON, L. (1988). Genes specifically expressed at growth arrest of mammalian cells. *Cell*, **54**, 787.

SINGH, S., ROSS, S.R., ACENA, M., ROWLY, D.A. & SCHREIBER, H. (1992). Stroma is critical for preventing or permitting immunological destruction of antigenic cancer cells. *J. Exp. Med.*, **175**, 139.

STANTON, L.W., WATT, R. & MARCU, K.B. (1983). Translocation, breakage and truncated transcripts of c-myc oncogene in murine plasmacytomas. *Nature*, **303**, 401.

TSO, J.Y., SUN, X., KAO, T., REECE, K.S. & WU, R. (1985). Isolation and characterization of rat and human glyceraldehyde-3-phosphate dehydrogenase cDNAs: genomic complexity and molecular evolution of the gene. *Nucleic Acids Res.*, **13**, 2485.